# ARTIFICIAL SELECTION ON RELATIVE BRAIN SIZE REVEALS A POSITIVE GENETIC CORRELATION BETWEEN BRAIN SIZE AND PROACTIVE PERSONALITY IN THE GUPPY

**DOI:** 10.1111/evo.12341

**Published:** 2014-02-01

**Authors:** Alexander Kotrschal, Eva JP Lievens, Josefin Dahlbom, Andreas Bundsen, Svetlana Semenova, Maria Sundvik, Alexei A Maklakov, Svante Winberg, Pertti Panula, Niclas Kolm, E Morrow

**Affiliations:** 1 Department for Integrative Biology and Evolution (KLIVV), Veterinary University ViennaSavoyenstrasse 1A, 1160 Vienna, Austria; 2 Department of Ecology & Genetics/Animal Ecology, Uppsala UniversityNorbyvägen 18D, SE-75236 Uppsala, Sweden; 4 Department of Neuroscience, Uppsala UniversityBox 593, SE-75124 Uppsala, Sweden; 5 Neuroscience Centre and Institute of Biomedicine/Anatomy, University of HelsinkiFIN-00014 Helsinki, Finland; 6 Department of Zoology/Ethology, Stockholm UniversitySvante Arrhenius väg 18B, SE-10691 Stockholm, Sweden

**Keywords:** Brain size, cognition, guppy, personality, *Poecilia reticulate*

## Abstract

Animal personalities range from individuals that are shy, cautious, and easily stressed (a “reactive” personality type) to individuals that are bold, innovative, and quick to learn novel tasks, but also prone to routine formation (a “proactive” personality type). Although personality differences should have important consequences for fitness, their underlying mechanisms remain poorly understood. Here, we investigated how genetic variation in brain size affects personality. We put selection lines of large- and small-brained guppies (*Poecilia reticulata*), with known differences in cognitive ability, through three standard personality assays. First, we found that large-brained animals were faster to habituate to, and more exploratory in, open field tests. Large-brained females were also bolder. Second, large-brained animals excreted less cortisol in a stressful situation (confinement). Third, large-brained animals were slower to feed from a novel food source, which we interpret as being caused by reduced behavioral flexibility rather than lack of innovation in the large-brained lines. Overall, the results point toward a more proactive personality type in large-brained animals. Thus, this study provides the first experimental evidence linking brain size and personality, an interaction that may affect important fitness-related aspects of ecology such as dispersal and niche exploration.

In humans and other animals alike, different individuals of the same species consistently show different behavioral and physiological reactions to similar contexts, and their behavior in one context is often predictive of their behavior in another. These sets of similar responses, which are termed animal personalities, behavioral syndromes (Sih et al. 2004b), coping styles (Koolhaas et al. 1999), or temperaments (Reale et al. 2000), refer to stable, long-term behavioral, emotional, and physiological differences in suites of traits among individuals of the same species (Carere and Locurto 2011). Here we use the broadest possible definition of animal personality and use the term to describe all the above phenomena (see Garamszegi and Herczeg 2012; Jandt et al. 2014).

Although animal personalities are believed to form a continuum (Sih et al. 2004b), much of the research in the field has focused on its endpoints. These endpoints are often referred to as proactive-reactive or bold-shy (Sih et al. 2004b) and there are many stereotypic differences in behavior between these personality types. For instance, proactive animals show a lower hypothalamic–pituitary–adrenal axis activity (they secrete lower levels of stress hormones in stressful situations [Korte et al. 1996]), exhibit higher levels of testosterone when compared to reactive individuals (Deruiter et al. 1992), are bolder and more exploratory in novel environments (Dingemanse et al. 2002), habituate more quickly to novel situations (Overli et al. 2005), are faster but less flexible learners (Coppens et al. 2010), and are more reluctant to break a learnt routine (Bolhuis et al. 2004). Such personalities have been repeatedly demonstrated in more than 100 species (Carere and Locurto 2011), ranging from ants (Wilson 1985) to primates (King and Figueredo 1997) and are thus ubiquitous in the animal kingdom. However, the factors underlying these consistent behavioral differences and driving the evolution of personality remain unclear.

Recently, Wolf et al. (2007) suggested an adaptive explanation for the evolution of risk taking, one aspect of animal personality related to proactive behavior. They based their argument on the trade-off between current and future reproduction, which often results in polymorphic populations, in which some individuals put more emphasis on current fitness returns whereas others put more emphasis on future fitness returns. Using life-history theory they then predicted that such differences in fitness expectations should result in systematic differences in risk-taking behavior. Although Wolf et al. offer a solution as to how extreme phenotypes (very risk-prone and very risk-averse) may evolve, it remains largely enigmatic how continuous variation over, and correlated responses between, multiple personality traits evolves.

Animal behavior is inevitably guided by neural processes in the brain; personality differences should therefore, almost by definition, result from differences in brain form and function. It is therefore reasonable to suppose that differences in brain size could play an important role in the formation of personality. The evolution of larger brains is suspected to be primarily driven by selection for increased cognitive ability (Jerison 1973; Dunbar 1998; Striedter 2005) and comparative and recent experimental evidence from a variety of taxa, including humans, suggests that larger brain sizes are associated with increased cognitive ability (Laughlin et al. 1998; van Schaik and Pradhan 2003; Tebbich and Bshary 2004; Isler and van Schaik 2006; Miller and Penke 2007; Niven and Laughlin 2008; Sol 2009; Brown 2012; Kotrschal et al. 2013a), while the costs of developing and maintaining large brains seem to limit brain size evolution (Aiello and Wheeler 1995; Kotrschal et al. 2013a). We hypothesize that individuals with larger brains use their greater cognitive abilities to perceive and integrate more information about their environment, or to respond more effectively to sudden changes in the environment. Such individuals should therefore behave differently from individuals that base their behavioral decisions on less information or that respond less effectively to a given situation. We adhere to the broad definition of cognition as comprising “all mechanisms that vertebrates have for taking in information through their senses, retaining it, and using it to adjust behavior” (Shettleworth 2010). Recent theoretical and empirical evidence suggests that personality type and cognition are functionally related (Overli et al. 2007; Sih and Del Giudice 2012) and that personality plays an important role in individual performance in tests of cognitive ability (Carere and Locurto 2011). In contrast, our hypothesis suggests a so far overlooked causality in the link between cognition and personality: that variation in brain size alters cognitive ability and thereby determines variation in personality.

If brain size selection induces changes in personality via increased cognition, we can derive several predictions from this hypothesis. First, we predict that individuals with larger brains can afford to behave more proactively in novel situations due to their increased ability to understand and/or respond to the new environment, and thus show a decreased stress response in those cases. We also expect them to outperform their conspecifics in learning a novel task (Lefebvre et al. 2004; Sol et al. 2005), which has recently been shown in guppies (females: Kotrschal et al. 2013a, males: Kotrschal et al. unpubl. ms.). These behavioral predictions for individuals with larger brains fit well with the descriptions of a proactive behavioral type (Koolhaas et al. 1999). We therefore hypothesize that an evolutionary increase in brain size shifts the population mean toward a more proactive behavioral type.

In this study, we test our “brain size drives personality” hypothesis through standard behavioral and physiological assays inspired by the above predictions, using recently developed replicated selection lines of large- and small-brained guppies (*Poecilia reticulata* [Kotrschal et al. 2013a]). As mentioned earlier, in these lines, large-brained females cognitively outperformed their smaller brained conspecifics in a test of numerical learning ability and large-brained males outperformed small-brained males in a test of spatial learning ability. We are therefore confident in our use of increased brain size as a proxy for greater cognitive abilities in the present study (see also Kotrschal et al. 2013b). To investigate the link between brain size and personality, we performed three sets of behavioral and physiological tests. First, to determine habituation, boldness, and exploration we observed individuals of different brain sizes in an open field test (Veenema et al. 2003). Second, to determine the physiological stress response we measured stress hormone release in a stressful situation (Overli et al. 2007). Finally, to investigate behavioral flexibility, which generally decreases with increased proactivity (Coppens et al. 2010), we quantified the response to a change in a familiar task, represented by a novel food source.

## Material and Methods

### THE BRAIN WEIGHT-SELECTED GUPPIES

We examined the relationship between cognitive ability and personality in laboratory populations of Trinidadian guppies (*P. reticulata*) that we artificially selected for large and small relative brain size (Kotrschal et al. 2012; Kotrschal et al. 2013a). The guppy has been a model organism for evolutionary biology for decades (Endler 1980; Reznick and Yang 1993; Bassar et al. 2013). The lines used here differ in relative brain size by 9% (Kotrschal et al. 2013a). Importantly, large-brained females tested for numerical learning ability outperformed small-brained females (Kotrschal et al. 2013a) and large-brained males outperformed small-brained males in a test of spatial learning (Kotrschal et al. unpubl. ms.).

All fish were housed in 3l aerated tanks containing 2 cm of gravel. The laboratory was maintained at 26°C with a 12:12 light:dark schedule. We minimized isolation stress by allowing visual contact between the tanks. Fish were fed flake food and freshly hatched brine shrimp six days per week. All behavioral experiments were done blindly because tanks were identified only by running numbers. We used several different groups of adult fish for our assays. The groups were balanced over the three replicate selection lines, the two brain size selection regimes and the two sexes. We used 72 fish for the open field test (during one week), 72 other individuals for the two tests of locomotive ability (two weeks), two sets of 72 fish for the test of physiological stress response (baseline and stress response measures [one week]), and a final 48 fish for the test of novel food acceptance (one week). We performed the assays in this sequence, spread over a period of four months.

### OPEN FIELD TEST

**Figure 1:**
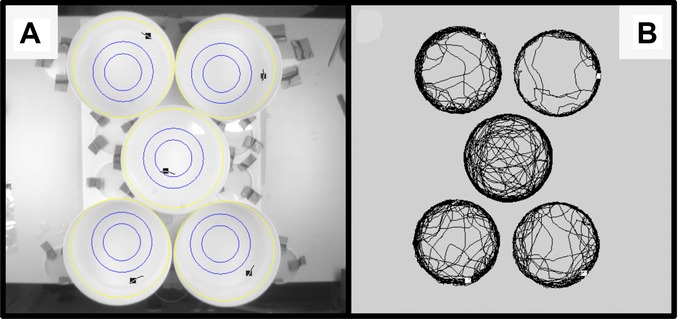
Setup to test spontaneous locomotion in an open field for adult guppies. (A) Yellow lines indicate the borders of each arena; blue lines indicate the borders between zones. The inner blue line denotes the border of the central zone used in the analysis of boldness. (B) Examples of swimming paths during the experiment. Note the clear differences in the way arena space is used by different individuals.

We tested locomotor behavior of guppies in an open field test. We gently deposited single fish into the center of opaque white, round testing arenas with a water depth of 4 cm and a diameter of 22 cm. Each trial was recorded for 15 min at 12.5 frames/sec by a digital video camera mounted 1.8 m above the arenas connected to a standard PC running the tracking software EthoVision Pro 3.1 (Noldus Information Technology, Wageningen, Netherlands), which logged coordinates of the fish for later analysis (Fig.1). To alleviate the effect of potential differences in handling and timing, the first minute of testing was not included (Burns 2008). EthoVision Pro 3.1 automatically calculated the parameters describing movement, and recorded when fish were present in the “outer” and “central” zones of the arena (Fig.1A). Of the recorded parameters, we regarded distance moved, meandering (absolute turning angle per centimeter swum [°/cm]), and time spent in the central zone as useful to quantify personality. First, we based our measure of “Habituation” on the rate of decrease in swimming distance over time. A faster decrease in swimming activity indicates faster habituation (Overli et al. 2005) because fish placed in novel environments are prone to “panic swimming,” that is, swimming very quickly to attempt escape from a threatening situation (which in this setup often manifests itself as a fast circling around the walls of the arena). “Exploration” was described by the rate of increase in meandering over time. We chose this proxy of exploration because, as opposed to panic swimming that is characterized by fast and linear movement, explorative swimming is slower, more “casual,” and follows more tortuous patterns. A higher turning angle per unit of distance covered is therefore indicative of more exploratory behavior (Sfakiotakis et al. 1999). Finally, “Boldness” was characterized as the amount of time fish spent in the open, unprotected central zone of the arena (in seconds), because thigmotaxis or “wall-hugging” is a common fear response in guppies (Warren and Callaghan 1976). Entering an open area increases the risk of attack by a predator; most fish therefore seek shelter and avoid open areas in unknown environments (Ostrander 2000). Thus, we considered individuals that stayed near the wall of the arena and away from the open zone to be more fearful. Every fish was tested once, limiting the biological independence of the variables. However, we stress that habituation, meandering, and boldness describe functionally different behaviors, and should thus be analyzed independently.

### SWIMMING PERFORMANCE

To exclude the possibility that any behavioral or exploratory differences in the open field test were a byproduct of the swimming ability of our experimental animals (e.g., as a consequence of energetic constraints; Aiello and Wheeler 1995), we quantified locomotive ability in two assays: anaerobic swimming performance (burst swimming speed; Hammer 1995) and aerobic swimming performance (critical swimming speed; Hammer 1995). To control for body size in these analyses, we determined standard length (SL, from the tip of the snout to the end of the caudal peduncle) to the nearest 0.5 mm on a standard 1 mm × 1 mm measuring board by eye, and weighed all individuals (after blotting them dry) to the nearest 1.0 mg after the assays.

**Figure 2:**
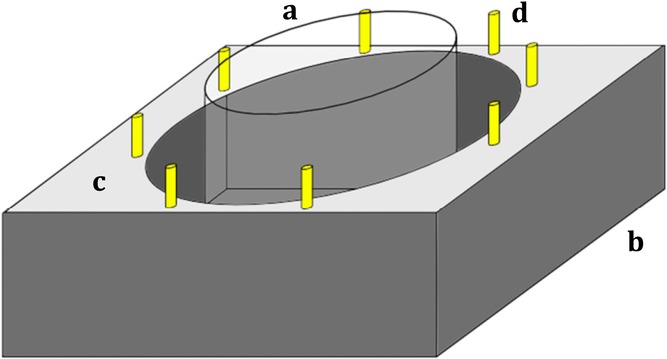
Experimental apparatus used to test burst-swimming speed. The setup consisted of a round transparent arena (a) set within an opaque tank (b), with a platform (c) serving as both a tipping point for the weights (d) and a shield against disturbances.

Burst speed is the initial maximal escape velocity of fish after a startle stimulus and indicative of escape success after a predator attack (Bailey and Batty 1984). We elicited and analyzed startle responses (“C-starts”; Domenici and Blake 1997) to investigate burst-swimming speed. As a testing arena (Fig.2), we placed a transparent Perspex ring (20 cm diameter, height 20 cm) in an opaque PVC fish tank (45 × 30 × 15 cm, water level 4 cm). Supported by the rim of the fish tank, a platform encircled the arena. The platform provided a standardized way to create startle-inducing stimuli: eight cylindrical metal weights (1.4 cm diameter, 5 cm high, weight 23 g) were placed evenly spaced around the rim of the platform from which they could easily be tipped into the fish tank. We placed each fish in the center of the testing arena and allowed it to acclimate for 10 min. We then tipped in the weight closest to the focal fish, which reliably elicited a startle response. The use of a transparent arena inside the fish tank allowed the fish to see the stimulus, without the procedure disturbing the water inside the arena itself. The test was filmed at 210 frames/sec by an Exilim EX-FH20 high-speed camera (Casio, Japan), mounted 85 cm above the arena. Each trial consisted of three tests, after each of which the fallen weight was replaced and the fish were allowed to recover for 2 min. We calculated burst-swimming speed from the video files based on the first 20 msec of the propulsive stroke of the C-start, which reliably captures the initial velocity of the fish (Odell et al. 2003). In a frame-by-frame analysis in Image J, we tracked the guppies’ center of mass and calculated the linear velocity from the displacement between first and last frame. The highest score of the three tests was considered the maximal burst speed. When comparing fish of different body sizes, swimming velocity is often expressed in body lengths per second (Domenici and Blake 1997). However, analyses using length-corrected velocity (SL/sec) yielded qualitatively similar results to absolute velocity (cm/sec) and as a result, we chose to show only the results for absolute velocity.

Critical swimming speed demonstrates an individual's capacity for endurance swimming, and is a direct indicator of its condition (Reidy et al. 2000). We measured the critical swimming speed of the experimental fish in a flow chamber, following Nicoletto (1991). The chamber consisted of a 115 cm long horizontally mounted transparent PVC pipe with an inner diameter of 1.8 cm. Aerated water was pumped into one end of the pipe, a diaphragm valve controlled flow rate and a rotameter allowed for accurate setting of flow rate. To assure rectilinear flow, a 6 cm collimator made from plastic straws of 5 mm diameter was placed at the inflow end of the pipe. We introduced fish into the chamber via an entry chute at the inflow end of the pipe. The opaque chute also provided a hiding area for the fish, which motivated them to swim near the inflow end. We measured critical swimming speed by subjecting fish to increased velocity tests. Individuals were forced to swim against a current, which was increased in discrete steps until exhaustion occurred and they were swept against the outflow end of the flow chamber. When a fish was put in the chamber, flow velocity was 0 cm/sec. A 10 min acclimation period at a low velocity of 6.5 cm/sec followed, a speed that guppies can maintain for several hours (Nicoletto 1996). Thereafter, we increased the current velocity by 2.2 cm/sec every 5 min (Oufiero and Garland 2009) until the fish reached exhaustion and was unable to detach itself from the mesh at the outflow end of the flow chamber for 3 sec. The current velocity at exhaustion and time until exhaustion were then used to calculate absolute critical speed in centimeter per second.

### PHYSIOLOGICAL STRESS RESPONSE

To quantify the cortisol stress response induced by a stressful situation, we placed each individual in 50 ml of holding water in white 400 ml beakers on a brightly lit workbench. Handling, exposure to bright lights without shelter and confinement in a relatively small body of water should all contribute to elicit a stress response. After 30 min, we euthanized the test subjects by cooling them quickly in ice water (<10 sec); we then removed the heads, shock-froze them in liquid nitrogen (<10 sec from ice water to liquid nitrogen), and stored the bodies at −80°C. For an estimate of the baseline cortisol levels of unstressed fish, we sacrificed another cohort directly after netting them from their individual holding tanks. Time between approaching the tank and euthanasia of fish was less than 10 sec. For cortisol analysis, we weighed the bodies to the nearest 0.1 mg and homogenized them in 1 ml of deionized water. We added 8 ml of ethyl acetate to the samples and shook them vigorously. The organic and aqueous phase was separated by a 3 min centrifugation where after the aqueous phase was frozen by placing the samples on dry ice. The organic phase was separated, transferred to a glass tube, and evaporated under a stream of N_2_ (g). The extraction procedure was repeated once and the organic phases pooled. To avoid residues on the tube walls, the glass tubes were rinsed with an additional 1 ml ethyl acetate, which was also evaporated under a stream of N_2_ (g). The cortisol was resuspended in phosphate buffered saline and cortisol was analyzed with a Salimetrics Saliva cortisol kit (Electra-box Diagnostica, Sweden; Cachat et al. 2010) according to the manufacturer's instructions. Calculations were made using a four-parameter logistic curve on www.readerfit.com.

### BEHAVIORAL FLEXIBILITY

To determine how readily individuals of different brain sizes adopt a novel food source, we placed the test subjects in individual 15 × 40 × 15 cm holding tanks with 1 cm of sand on the bottom and constant aeration. In these tanks, we prevented visual contact between fish to exclude social learning from watching other individuals. The familiar food types in these populations were flake food and brine shrimp, and the animals were therefore used to feeding from the surface or water column. As a novel food source we used commercially available food pellets (Sera O-nip, Aquafoods Inc.), which quickly sank to the bottom and had to be actively nibbled on to release bits of food. We dropped one-quarter of a pellet randomly on one side of the tank and observed each focal individual for 2 min directly afterwards. After 4 h we checked whether feeding had occurred, and removed all uneaten food from the tank. The pellets disintegrated over this time span and formed small conical mounds of food. If this mound was reduced in size, scattered, or gone we concluded that feeding had occurred. Together with the direct observation of feeding behavior, this provided an accurate assessment of whether feeding had occurred or not. We offered the food pellets once per day for seven consecutive days.

### STATISTICAL ANALYSES

We analyzed all movement experiments using linear mixed-effects models in R (R Development Core Team 2006). Fixed effects, where relevant, were selection regime (large or small brain size), sex, a sex/selection regime interaction, SL, and water temperature. Replicate selection line (three lines with one upselected and one downselected population each) was a random factor in all analyses; for the open field analyses we nested an individual term within it. Tracking data from the open field test was summed over 20 sec intervals, limiting noise in the data, and these time points were taken as a covariate in our analyses. We performed hypothesis testing using the likelihood ratio test (Zuur et al. 2009). To describe swimming performance, we tested for brain size-dependent effects on critical and burst speed. To analyze movement behavior in a novel environment, we tested for brain size and sex effects on habituation (rate of decrease in swimming distance), exploration (rate of increase in meandering), and boldness (rate of increase in time spent in the central zone). In addition, we tested for personality by calculating the significance of the random term “individual” (Herczeg et al. 2013). Note that, for the data recorded in the open field test, the sheer size of the dataset prevented residual distributions from being perfectly normal despite transformation; they were always biased toward more central estimates.

To analyze the physiological stress response toward confinement in an open space we used a linear mixed-effect model with cortisol concentration as the dependent variable. We included selection regime and sex as fixed factors, replicate line as a random factor, and body weight as a covariate. In preliminary models we tested all possible interaction terms between factors, and between factors and the covariate. Because these interaction terms were not significant, we excluded them from the final model. Due to sample losses we analyzed 118 specimens (64 from the stressed and 54 from the control group). These analyses were based on F statistics and performed in SPSS 19.0 (SPSS Inc., Chicago, IL).

To analyze how readily individuals of different brain sizes accept a novel food source, we used a generalized linear mixed-effect model in R, with the probability of feeding on the food pellet as the dependent variable. We included selection regime and sex as fixed factors and individual nested within replicate line as random factors. We used likelihood ratio tests to test all possible interaction terms between factors, and between factors and the covariate. Nonsignificant terms were excluded from the final model. We also tested for personality by calculating the significance of the random term “individual” in the final model.

Data is deposited in the Dryad digital repository.

## Results

**Figure 3:**
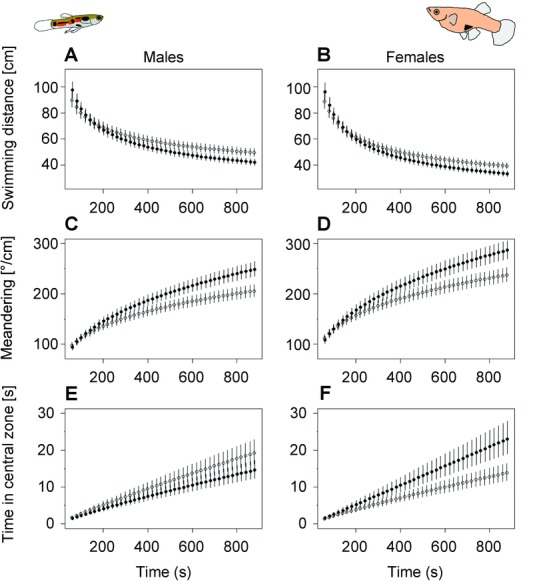
Behavioral changes of large-brained (filled circles) and small-brained (open circles) individuals over time in a novel environment (males left panel, females right panel), as predicted by the optimal models. Depicted is (A, B) the decrease in swimming distance for large- and small-brained individuals (*P* < 0.0001), (C, D) the increase in meandering (*P* < 0.0001), and (E, F) the increase in time spent in the central zone (females: *P* < 0.0001).

In the open field test, we found that the behavior of large-brained individuals differed markedly from that of small-brained individuals. First, although all fish became habituated to the novel environment (the distance swum decreased significantly with time for all fish, *P* < 0.0001), large-brained individuals showed faster habituation. In both males and females, large-brained guppies reduced their swimming activity faster than small-brained guppies (*P* < 0.0001, Table1, Fig.3A). Second, explorative behavior also increased more quickly in large-brained over small-brained individuals. All fish increased their rate of meandering over time (*P* < 0.0001), thus becoming more explorative, but the rate of change was faster in both male and female large-brained individuals (*P* < 0.0001, Table1, Fig.3B). For our measure of boldness, provided by time spent in the central zone, we found a sex-specific response. Large-brained females spent more time in the central zone as compared to small-brained females (*P* < 0.0001, Table1, Fig.3C), while males of different brain sizes did not differ in the time spent in the central zone. For all three variables, we found a significant effect of the random term “individual,” indicating that there were consistent interindividual differences, that is, personality differences (all *P* < 0.0001, Table1).

**Table 1:** Summaries of the optimal models for tests of different aspects of personality (habituation, exploration, boldness, behavioral flexibility) in guppies with different brain sizes

Parameter	Fixed effect	Effect size	SE	df	*P*
Habituation (distance swum)					
[log(cm)]	log(time)	−0.30	0.01	2936	**<0. 0001**
	log(time): brain size (large)	−0.09	0.02	2936	**<0. 0001**
	log(time): sex (male)	0.08	0.02	2936	**<0. 0001**
Random effects: replicate, individual nested within replicate (***P* < 0.0001**)					
Exploration (meandering)					
[log(°/cm)]	log(time)	0.28	0.01	2937	**<0. 0001**
	log(time): brain size (large)	0.08	0.02	2937	**<0. 0001**
Random effects: replicate, individual nested within replicate (***P* < 0.0001**)					
Boldness (time spent in central zone)					
[log(sec)]	log(time)	0.86	0.02	2935	**<0.0001**
	log(time): brain size (large)	0.14	0.03	2935	**<0.0001**
	log(time): sex (male)	0.04	0.03	2935	0.22
	log(time): brain size (large): sex (male)	−0.19	0.05	2935	**0.0001**
Random effects: replicate, individual nested within replicate (***P* < 0.0001**)					
Behavioral flexibility (probability of feeding)					
[logit(feeding)]	Intercept	2.23	0.34		**<0.0001**
	Brain size (large)	−0.94	0.35		**0.007**
	Sex (male)	−2.56	0.35		**<0.0001**
Random effects: replicate, individual nested within replicate (***P* = 0.02**)					

Analyses of the open field test were conducted with a focus on time (included as a covariate), accordingly intercept values are not provided. Model selection followed Zuur et al. (2009). Statistically significant results (*P* < 0.05 based on likelihood ratio tests) are highlighted in bold.

In our tests of swimming ability, absolute burst speed was positively influenced by body size but independent of sex or brain size (Table2). Critical swimming speed was also independent of brain size (Table2). Instead, critical swimming speed was dependent on body size, water temperature, and sex (Table2).

**Table 2:** Summaries of the optimal models for tests of different aspects of locomotor ability (burst speed, critical speed) in guppies with different brain size

Parameter	Fixed effect	Effect size	SE	df	*P*
Burst speed					
[log(cm/sec)]	Body size	−1.44	0.42	68	**0.02**
Random effects: replicate					
Critical speed					
[log(cm/sec)]	Body size	−0.35	0.31	64	0.27
	Temperature	0.12	0.05	64	**0.03**
	Sex (male)	−3.59	1.33	64	**0.01**
	Body size: sex (male)	2.03	0.77	64	**0.01**
Random effects: replicate					

Note that the factors “body size”, “temperature”, and “sex” were only included to control for variation in swimming performance and will not be discussed further.

Model selection followed Zuur et al. (2009). Statistically significant results (*P* < 0.05 based on likelihood ratio tests) are highlighted in bold.

**Figure 4:**
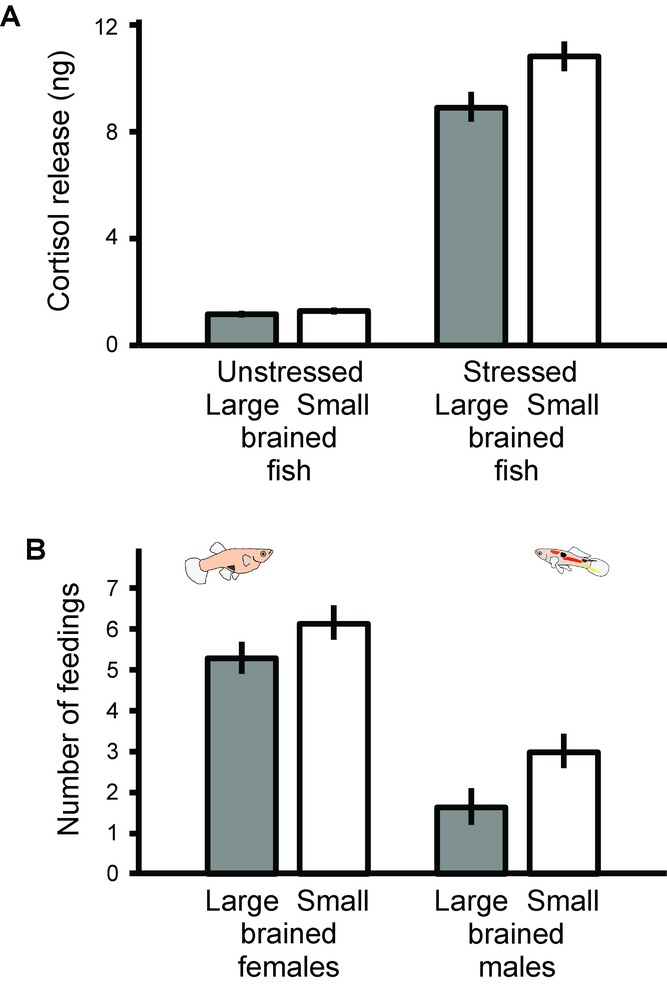
Individuals selected for small and large relative brain size differ in stress response and behavioral flexibility. (A) Stress response to confinement. Depicted is the mean concentration per gram body mass (±SEM) of whole-body cortisol extracts for large- and small-brained individuals unstressed (*P* = 0.455) and after confinement (*P* = 0.020). (B) Propensity to feed on a novel food source. Depicted is the mean number of times (out of eight times; ±SEM) the animals fed from the offered pellet (small-brained individuals fed more often; *P* = 0.020). Both graphs show the estimated marginal means derived from the GLMM described in the main text.

In our stress test, we found that independently of sex, large-brained individuals released less cortisol in this stressful situation (GLMM: brain size: *F* = 5.73, *P* = 0.020, df = 58.1; sex: *F* = 1.57, *P* = 0.215, df = 58.1; replicate: *F* = 0.42, *P* = 0.660, df = 58.2; body weight: *F* = 46.41, *P* < 0.001, Fig.4A). Baseline cortisol levels did not differ between groups (GLMM: brain size: *F* = 0.567, *P* = 0.455, df = 49.1; sex: *F* = 3.978, *P* = 0.052, df = 49.1; replicate: *F* = 4.861, *P* = 0.012, df = 49.2; body weight: *F* = 0.146, *P* = 0.704).

Finally, when presented with a novel food type, brain size and sex had a significant effect on the probability to feed. Females and small-brained individuals were more likely to feed on the novel food than males and large-brained individuals (*P* < 0.001 and *P* < 0.01, respectively; Table1, Fig.4A), and there were significant among-individual differences (*P* = 0.020 for the random factor “individual,” Table1).

## Discussion

In our experiment, strong directional selection on relative brain size led to correlated responses in multiple aspects of animal personality. Large-brained individuals were faster to habituate to and more explorative in a novel environment, and demonstrated higher routine fidelity when adjusting to a novel food source. Both these aspects are characteristic of more proactive personalities (Koolhaas et al. 1999; Sih et al. 2004a). Furthermore, they had a reduced cortisol stress response, a trait that is also typical of proactive individuals (Overli et al. 2007). We therefore suggest that the results are congruent and conjointly imply that large-brained individuals have a more proactive personality.

We advocate here that the selection on brain size, which increased cognitive ability in both males and females, enabled the expression of more proactive behavior in large-brained individuals. Although our data only allow us to speculate about the mechanism(s) that generate the association between brain size and personality, we propose that individuals with larger brains and therefore greater cognitive ability require less time to assess a novel environment, thus habituating more quickly. This effect could either be caused by a more accurate assessment of potential threats or a greater ability to respond to those threats.

Translated to the natural environment of the guppy, increased cognition and proactivity would provide several benefits to large-brained fish. First, individuals with lower cognitive abilities would be forced to forage close to cover, whereas individuals with greater cognitive abilities and more accurate knowledge of the environment could also use more open areas. Large-brained individuals should therefore be more prone to disperse and/or explore novel niches. Indeed, bold great tits (*Parus major*; Dingemanse et al. 2003), lizards (*Lacerta vivipara*; Cote and Clobert 2007), and mosquitofish (*Gambusia holbrooki*; Cote et al. 2010) are known to disperse more than their shy conspecifics. Second, cognitive abilities may also be highly important when under direct threat from predators. In a previous experiment on guppies, more active, bold, and exploratory fish were found to survive longer when exposed to a predator (Smith and Blumstein 2010). Third, guppy females are known to preferentially mate with bold males (Godin and Dugatkin 1996). Thus, for males, larger brains may confer additional fitness benefits by increasing their mating success. These potential benefits of larger brains raise the question of what limits brain size evolution. Likely candidates include the prohibitively high-energy requirements and reproductive costs of neural development and maintenance (Aiello and Wheeler 1995; Kotrschal et al. 2013a),

In the open field test, large-brained individuals swam less and meandered more than small-brained individuals. In line with our argument above, we attribute these differences in activity to differences in cognitive abilities. Because high-cognition individuals are quicker to assess the relative safety of a novel situation and/or have greater means to deal with the “unexpected” (Sol 2009; Kotrschal and Taborsky 2010), they may also be able to habituate more quickly. This may explain the general pattern of faster habituation in proactive compared to reactive animals in other taxa (Overli et al. 2005). We suggest that the higher meandering was a correlated response from the more explorative tendencies of large-brained individuals. Because we did not find significant relationships between our tests of locomotive ability (burst speed and critical speed) and brain size, we conclude that the more exploratory behavior and faster reduction in swimming activity of large-brained animals cannot be attributed to differences in locomotive ability per se.

In the open field test, large-brained females were also bolder than small-brained females. This boldness measure was based on the amount of time the fish spent in the open central zone of the testing arena: large-brained fish used the whole arena whereas small-brained fish spent more time close to the sides of the arena and avoided the central exposed zone. We did not find differences for large- and small-brained males, a result that may be caused by sex-based differences in ecology. Guppy females usually have limited home ranges, which they rarely leave, whereas males explore much larger areas and frequently cross large open spaces (Croft et al. 2003). It is therefore feasible that the arenas used, while adequate for investigating female space use, were too small to assess male space use. Future tests with larger arenas should verify this suggestion.

Next, we tested cortisol production in response to a stressor. When challenged, animals initially display an active behavioral response, sustained by an immediate activation of the sympatheticochromaffin system, which induces a rapid mobilization of stored energy substrates. In contrast, loss of behavioral control typically results in a switch to a passive freezing response associated with behavioral inhibition and elevated corticosterone secretion. Animals with a proactive stress coping style are characterized by retaining the active behavioral response, including behavioral control with active avoidance, and showing a drastic elevation in circulating cathecholamines but only modest elevations of plasma corticosteroid levels (Koolhaas et al. 1999). We found that large-brained individuals had lower cortisol levels under confinement, a difference in physiological stress response that could be driven by differences in stress-coping strategies between individuals with small and large brains. Animals with larger brains are generally better at devising novel or flexible solutions (Sol 2009), and should thus maintain a greater behavioral control in response to a stressor. In colloquial terms, they quickly come to terms with situations that are not immediately threatening, while individuals with smaller brains remain stressed. This is further corroborated by the faster decrease in movement in large-brained fish. In human cognitive sciences, this potential “cognitive control over emotion” has been identified as a key prerequisite of the evolution of higher cognitive abilities (Ochsner and Gross 2005).

As a final assessment of how brain size may drive personality, we investigated behavioral flexibility in the two selection lines. Lower behavioral flexibility, or routine fidelity, is a key feature of a bold and proactive personality type (Sih et al. 2004b, Coppens et al. 2010). When we provided a never-before-encountered food type, we found that large-brained individuals were less likely to feed from the novel food type in both sexes. We suggest that individual fish had formed a routine regarding the place and mode of feeding prior to the change in food type. Small-brained individuals were faster to break this routine and accept the novel food source, whereas the large-brained individuals upheld their routine longer. Similar results have been demonstrated in a study on rainbow trout, which reported that animals selected for low cortisol stress reactions were bolder and less behaviorally flexible than animals selected for high cortisol stress reactions (Ruiz-Gomez et al. 2011). A similar study in mice and rats found that socially bold individuals formed routines in a maze, whereas more timid individuals retained flexibility (Benus et al. 1987). Bold barnacle geese also form feeding routines quickly, whereas shy geese continue using social information in their feeding decisions (Kurvers et al. 2010). It is puzzling that animals with larger brains should be less responsive to novel challenges, because a more flexible structure of behavior (Sol 2009) seems intuitively advantageous. One explanation is that individuals with larger brains may be better at allocating their “computing capacity.” Because a constantly reoccurring situation requires little capacity once a solution is devised, the decision to form routines should free cognitive capacity for other tasks. Selective allocation of cognitive capacity is known from functional MRI scanning studies of classical conditioning in humans (Dawson et al. 1982). Large-brained individuals may thereby trade-off behavioral flexibility for an even higher cognitive capacity. Because faster routine formation and higher routine fidelity are key features of a bold and proactive personality type (Sih et al. 2004b; Wolf et al. 2008), the greater responsiveness toward novel food in small-brained individuals further corroborate our conclusion that a larger brain size creates a more proactive personality type. In addition to the brain size difference in acceptance of the novel food, females were quicker to start feeding than males. This was expected because in guppies, females are more active and innovative while foraging (Laland and Reader 1999), most likely reflecting the fact that female reproductive success is mainly food-limited, whereas males are more limited by their access to females (Houde 1997).

To conclude, our results demonstrate that variation in relative brain size directly affects animal personality, and that large-brained guppies display a remarkably close match in their behavior to what is expected in proactive individuals (Sih et al. 2004b). We propose that it is the difference in cognitive abilities, which changes the qualitative and quantitative awareness of the surrounding environment, and/or generates a greater ability to deal with the unexpected, that opens up the possibility for more proactive behavior in large-brained individuals. Future analyses will focus on disentangling these two possibilities.

The experiments were done in accordance with the ethical regulations for research involving animal subjects in Uppsala, Sweden, under the permit C50/12.

## DATA ARCHIVING

The doi for our data is 10.5061/dryad.6gd87.
